# Efficacy and Adverse Events of Apatinib Salvage Treatment for Refractory Diffuse Malignant Peritoneal Mesothelioma: A Pilot Study

**DOI:** 10.3389/fonc.2022.811800

**Published:** 2022-07-01

**Authors:** Zhi-Ran Yang, Yan-Dong Su, Ru Ma, He-Liang Wu, Yan Li

**Affiliations:** ^1^ Department of Peritoneal Cancer Surgery, Beijing Shijitan Hospital, Capital Medical University, Beijing, China; ^2^ Department of Peritoneal Cancer Surgery, Beijing Shijitan Hospital, Peking University Ninth School of Clinical Medicine, Beijing, China

**Keywords:** malignant peritoneal mesothelioma, apatinib, disease control rate, tumor makers, adverse events

## Abstract

**Objective:**

To investigate the clinical efficacy and adverse events (AEs) of apatinib salvage treatment for diffuse malignant peritoneal mesothelioma (DMPM) that has failed to respond to the recommended treatments.

**Methods:**

27 patients with refractory DMPM were treated with apatinib at our center from April 2014 to October 2020, at the initial dose of 250 mg/d. The dose was reduced to 125 mg/d when serious adverse events (SAEs) occurred. 28-day was set as a treatment cycle. The frequency of follow up was once every 28 days. The efficacy evaluation was conducted according to the Response Evaluation Criteria in Solid Tumors (RECIST) 1.1 criteria and the serum tumor markers before and after apatinib treatment. The safety assessment was performed with the National Cancer Institute (NCI) Common Terminology Criteria for Adverse Events (CTCAE) version 5.0. The primary endpoints were objective response rate (ORR) and disease control rate (DCR), and the secondary endpoints were AEs.

**Results:**

The 27 patients completed a median treatment-cycle of 15.0, ranging from 5.1 to 39.4 cycles. At the median follow-up of 14.3 (4.8-51.8) months, median overall survival (OS) was 59.4 months, median apatinib-treatment-related survival (ATRS) was 14.0 (4.8-36.8) months. Complete response (CR) was observed in 0 case (0.0%), partial response (PR) in 4 cases (14.8%), stable disease (SD) in 12 cases (44.4%), and progression disease (PD) in 11 cases (40.7%). The ORR was 14.8%, and DCR was 59.3%. The median serum CA125 values before and after apatinib treatment were 32.9 (7.0-4592.4) U/mL and 29.7 (6.1-4327.4) U/mL, respectively (*P*=0.009). The common AEs were hypertension (6/27; 22.2%), hand-foot syndrome (5/27; 18.5%), albuminuria (4/27; 14.8%), anemia (4/27; 14.8%), leukopenia (4/27; 14.8%), rash (2/27; 7.4%), fatigue (2/27; 7.4%), oral ulcers (2/27; 7.4%), hoarseness (2/27; 7.4%), nausea/vomiting (2/27; 7.4%), diarrhea (2/27; 7.4%), headache (1/27; 3.7%), and fever (1/27; 3.7%). The incidence rate of grade III/IV AEs was 16.2%.

**Conclusions:**

Apatinib is effective in treating refractory DMPM, with promising efficacy and acceptable safety.

## Introduction

Diffuse malignant peritoneal mesothelioma (DMPM) is a rare malignancy characterized by highly malignant behavior and poor prognosis (1). The median overall survival (OS) of the patients was about 4-13 months treated by traditional treatment strategies, such as palliative surgery, intravenous and intraperitoneal chemotherapy (2, 3). The implementation of cytoreductive surgery (CRS) and hyperthermic intraperitoneal chemotherapy (HIPEC) dramatically prolonged the OS of DMPM to 30-92 months, and a meta-analysis of 20 publications with data on outcomes of 1047 patients with DMPM treated with CRS and HIPEC reported a 5-year survival of 42% (1, 4–6). However, postoperative recurrence is still an important cause of death for most patients, and some still showed no benefits from CRS and HIPEC (7). Given the rarity of this malignancy, there have been few randomized trials. Thus, new therapies are in urgent need to improve the prognosis of DMPM patients.

Neovascularization plays an important role in the growth and metastasis of malignant tumors. Vascular endothelial growth factor (VEGF) and its receptor vascular endothelial growth factor receptor-2 (VEGFR-2) were overexpressed in DMPM, suggesting that VEGF and VEGFR-2 may be a potential target for the treatment of DMPM (8).

Apatinib is a molecular targeted drug that selectively targets the VEGFR-2, and blocks the downstream signal transduction to inhibit neovascularization. Apatinib has been approved for patients with advanced gastric cancer/gastric-esophageal junction cancer (9, 10). Some studies have also shown that apatinib could directly inhibit the proliferation, invasion or metastasis of ovarian cancer, hepatobiliary cancer and other tumor cells by inhibiting the PI3K/AKT signaling pathway and epithelial-mesenchymal transition (11–13). Moreover, the PI3K/AKT signaling pathway was abnormally activated in DMPM cells (14). Therefore, apatinib could have the treatment potential for DMPM.

Our experimental studies have demonstrated the effectiveness of apatinib on DMPM (15, 16). Therefore, this pilot study was conducted to explore the safety and efficacy of apatinib for refractory DMPM patients, thus providing clinical evidence for the application of apatinib.

## Patients and Methods

### Clinical Information

This study included 27 patients with DMPM who received apatinib treatment at our center from April 2014 to October 2020. Complete information was collected including clinical pathological characteristics, treatment regimens, efficacy evaluation, adverse events, and follow-up information. The study protocol was approved by the institutional review board and the ethics committee, all the patients signed the informed consent.

Major inclusion criteria were: (1) DMPM diagnosis was confirmed by histopathology, and thoracic-abdominal-pelvic computed tomography (CT); (2) age ≥ 18 years; (3) Karnofsky performance status (KPS) score ≥ 60 and the expected survival ≥ 3 months; (4) patients progressed or relapsed after pemetrexed/gemcitabine combined with platinum chemotherapy; (5) patients received other anti-angiogenic agent for at least 3 months; (6) at least a lesion can be measured; (7) acceptable liver and renal function, with blood total bilirubin level ≤ 1.5 × the upper limit of normal (ULN), alanine transaminase and aspartate transaminase ≤ 5 × ULN, and serum creatinine ≤ 1.25 × ULN.

Major exclusion criteria were: (1) patients who developed grade III-V serious adverse events (SAEs) after chemotherapy or delayed surgical wound healing after surgery; (2) patients who had abnormal coagulation function, with international normalized ratio > 1.5 or prothrombin time > ULN + 4 s, or had obvious gastrointestinal bleeding; (3) patients who had drug-resistant hypertension or abnormal urine protein; (4) patients who could not take medicine orally; (5) patients with severe major organ dysfunctions; (6) women at pregnancy or breastfeeding; (7) patients with mental illness.

### Treatment Protocol

**Figure 1:**
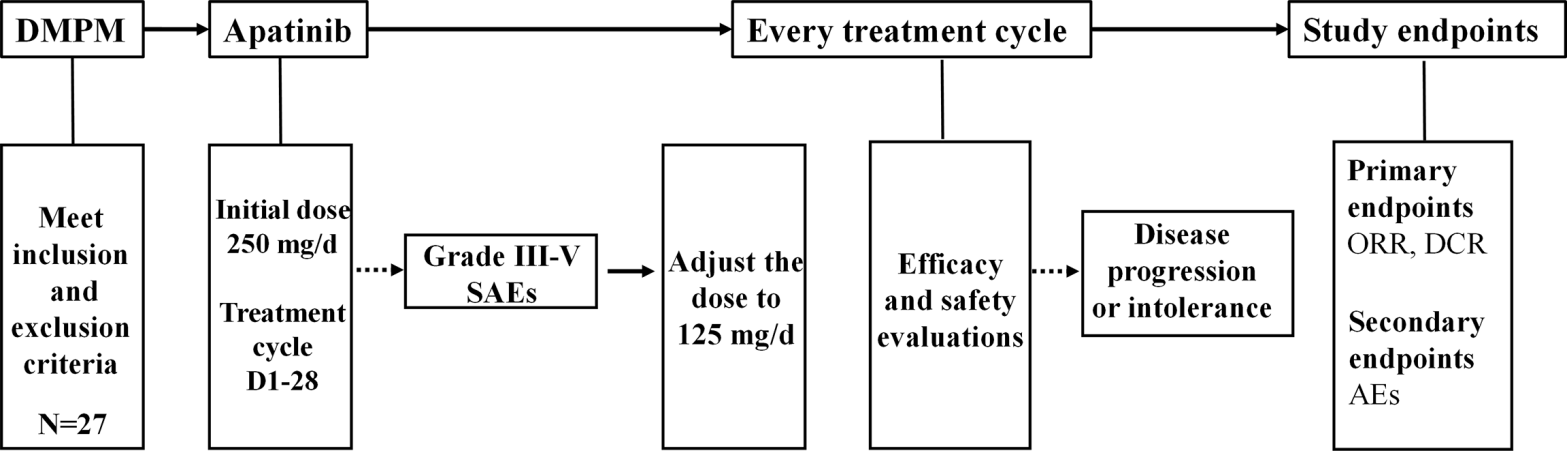
The flow chart of apatinib treatment for DMPM. DMPM, diffuse malignant peritoneal mesothelioma; SAEs, serious adverse events; ORR, objective response rate; DCR, disease control rate; AEs, adverse events.

Apatinib was provided by Jiangsu Hengrui Medicine Co., Ltd. (Nantong, Jiangsu, China), and administered orally daily. Patients were treated with apatinib at the initial dose of 250 mg daily, and reduced to 125 mg when SAEs occurred until disease progression or intolerance. 28-day was set as a treatment cycle. The efficacy and safety were evaluated after the patients completed at least one treatment cycle. The flow chart of apatinib treatment for DMPM was shown in **Figure 1**. All treatment-related information was monitored and recorded, including the performance status, symptoms, physical examination, blood pressure, complete blood count, urine tests, liver function, kidney function, tumor markers and the computed tomography (CT).

### Efficacy and Safety Evaluations

The apatinib efficacy evaluation was conducted according to the Response Evaluation Criteria in Solid Tumors (RECIST) 1.1 criteria, including complete response (CR), partial response (PR), stable disease (SD), and progressive disease (PD). Serum tumor markers were also monitored before and after apatinib treatment, including the carbohydrate antigen 125 (CA125), carbohydrate antigen 199 (CA199) and carcinoembryonic antigen (CEA). Before apatinib was defined as within the 3 days of apatinib treatment, and after apatinib was referred to the most recent serum tumor markers before the follow-up date, or after the last cycle for those who obtained PD. The primary endpoints of this study were objective response rate (ORR) and disease control rate (DCR), and the secondary endpoints were adverse events (AEs).

The National Cancer Institute (NCI) Common Terminology Criteria for Adverse Events (CTCAE) version 5.0. was used to evaluated the safety of apatinib treatment. The severity of AEs was divided into 5 grades. Detailed criteria for these AEs were the following. Grade I Mild: asymptomatic or mild symptoms; clinical or diagnostic observations only; intervention not indicated. Grade II Moderate: minimal, local or noninvasive intervention indicated; limiting age-appropriate instrumental activities of daily living (ADL). Grade III Severe or medically significant but not immediately life-threatening: hospitalization or prolongation of hospitalization indicated; disabling; limiting self-care ADL. Grade IV Life-threatening consequence: urgent intervention indicated. Grade V Death related to AEs. SAEs were defined as the grades III-V AEs.

### Follow-up

The frequency of follow up was once every 28 days. The follow-up consisted of performance status, efficacy response assessment, AEs, laboratory examination, medical imaging examination if necessary, and survival information of the patients. The last follow-up by telephone or outpatient clinic was on October 30, 2021, with 100% follow-up rate. The overall survival (OS) was defined as the time interval from disease diagnosis to death or the last follow up. The apatinib-treatment-related survival (ATRS) was defined as the time interval from apatinib treatment to death, treatment discontinuation, or the last follow-up.

### Statistical Analysis

Microsoft Excel 2013 and Statistical Package for Social Science 22.0 (SPSS 22.0, IBM Corporation, SPSS, Armonk, NY) software were used for data analysis. GraphPad Prism 8.0.1 (GraphPad, San Diego, USA) was used for image processing. Non-parametric tests were used to analyze the differences of serum tumor markers before and after treatment. *P*<0.05 was considered as statistically significant.

## Results

### Major Clinicopathological Characteristics of the Patients in This Study

A total of 27 patients with refractory DMPM were enrolled, including 10 males (37.0%) and 17 females (63.0%), with a median age of 56 (24-77) years, and 3 cases (11.1%) with a history of asbestos exposure. In terms of pathology, there were 24 cases (88.9%) with epithelioid type, 2 cases (7.4%) with biphasic type, and 1 case (3.7%) with sarcomatoid type. There were 8 patients (29.6%) previous treated with CRS+HIPEC, and 19 patients (70.4%) previously treated with pemetrexed/gemcitabine combined with platinum chemotherapy. The median ATRS was 14.0 (4.8-36.8) months (**Table 1**).

**Table 1:** Major clinicopathological characteristics of 27 patients with refractory.

Characteristics	No. of patients
Gender, n (%)	
Male	10 (37.0)
Female	17 (63.0)
Age (years), median (range)	56 (24-77)
CRS+HIPEC	
Yes	8 (29.6)
No	19 (70.4)
History of chemotherapy, n (%)	
Yes	19 (70.4)
No	8 (29.6)
History of asbestos exposure, n (%)	
Yes	3 (11.1)
No	24 (88.9)
Histopathological type, n (%)	
Epithelioid type	24 (88.9)
Biphasic type	2 (7.4)
Sarcomatoid type	1 (3.7)
The median ATRS (months) (range)	14.0 (4.8-36.8)

DMPM, diffuse malignant peritoneal mesothelioma; CRS, cytoreductive surgery; HIPEC, hyperthermic intraperitoneal chemotherapy; ATRS, apatinib-treatment-related survival; No., number.

### Efficacy of Apatinib Treatment

The 27 DMPM patients treated for at least 1 cycle of apatinib were included in the efficacy analyses. At a median follow-up of 14.3 (4.8-51.8) months, no CR was observed, 4 patients (14.8%) showed PR, 12 patients (44.4%) had SD, 11 patients (40.7%) had PD. The ORR was 14.8%. The DCR was 59.3% (**Table 2**).

**Table 2:** Efficacy of apatinib in the treatment of 27 DMPM patients.

Efficacy	No. of patients	No. (%)
CR	0	0.0
PR	4	14.8
SD	12	44.4
PD	11	40.7
ORR	4	14.8
DCR	16	59.3

DMPM, diffuse malignant peritoneal mesothelioma; CR, complete response; PR, partial response; SD, stable disease; PD, progressive disease; ORR, objective response rate; DCR, disease control rate; No., number.

### Evaluation of the Serum Tumor Markers

**Figure 2:**
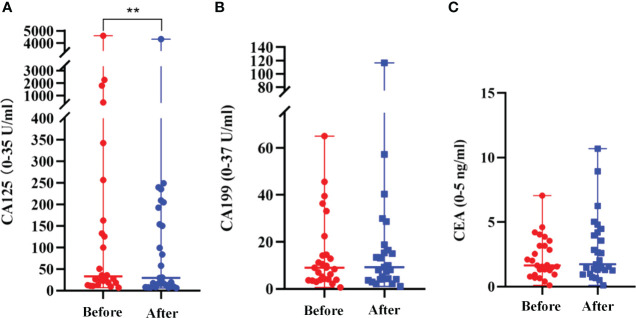
Changes of the serum tumor marker. **(A)** Serum CA125 values before and after apatinib treatment (**P<0.01); **(B)** Serum CA199 values before and after apatinib treatment (*P*=0.499); **(C)** CEA values before and after the apatinib treatment (*P*=0.085).

The median serum CA125 values before and after apatinib treatments were 32.9 (7.0-4592.4) U/mL and 29.7 (6.1-4327.4) U/mL, respectively (*P*=0.009); the median serum CA199 values before and after treatment were 9.0 (0.6-65.0) U/mL and 9.2 (1.1-116.76) U/mL, respectively (*P*=0.499); and the median serum CEA values before and after the treatment were 1.7 (0.1-7.1) ng/mL and 1.7 (0.1-10.7) ng/mL, respectively (*P*=0.085) (**Figure 2** **;** **Table 3**).

**Table 3:** Evaluation of the serum tumor markers before and after apatinib treatment.

Serum tumor markers	Groups		P
	Before the treatment	After the treatment	
CA125 (U/mL)	32.9 (7.0-4592.4)	29.7 (6.1-4327.4)	0.009
CA199 (U/mL)	9.0 (0.6-65.0)	9.2 (1.1-116.76)	0.499
CEA (ng/mL)	1.7 (0.1-7.1)	1.7 (0.1-10.7)	0.085

### Survival Analysis

**Figure 3:**
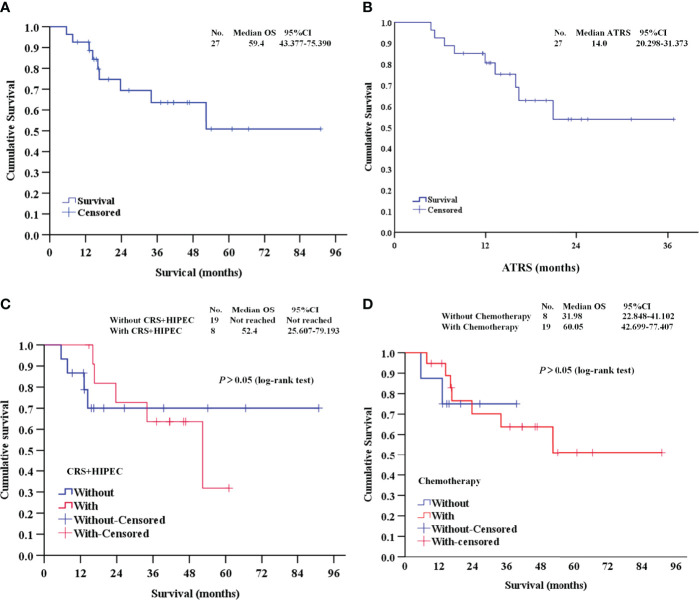
Survival analysis of this study. **(A, B)** The OS and ATRS of the 27 DMPM patients; **(C)** Survival analysis between patients treated with vs. without CRS+HIPEC; **(D)** Survival analysis between patients treated with vs. without previous chemotherapy. OS, overall survival; ATRS, apatinib-treatment-related survival; CRS+HIPEC, cytoreductive surgery + hyperthermic intraperitoneal chemotherapy.

At the median follow-up of 14.3 months (range 4.8-51.8), 9 patients (33.3%) died, and 18 patients (66.7%) survived. The median OS was 59.4 months (**Figure 3A**). The median ATRS was 14.0 months (**Figure 3B**). There were no statistically significant differences in OS between patients treated with vs. without CRS+HIPEC (**Figure 3C**), and between patients treated with vs. without previous chemotherapy (**Figure 3D**).

**Figure 4:**
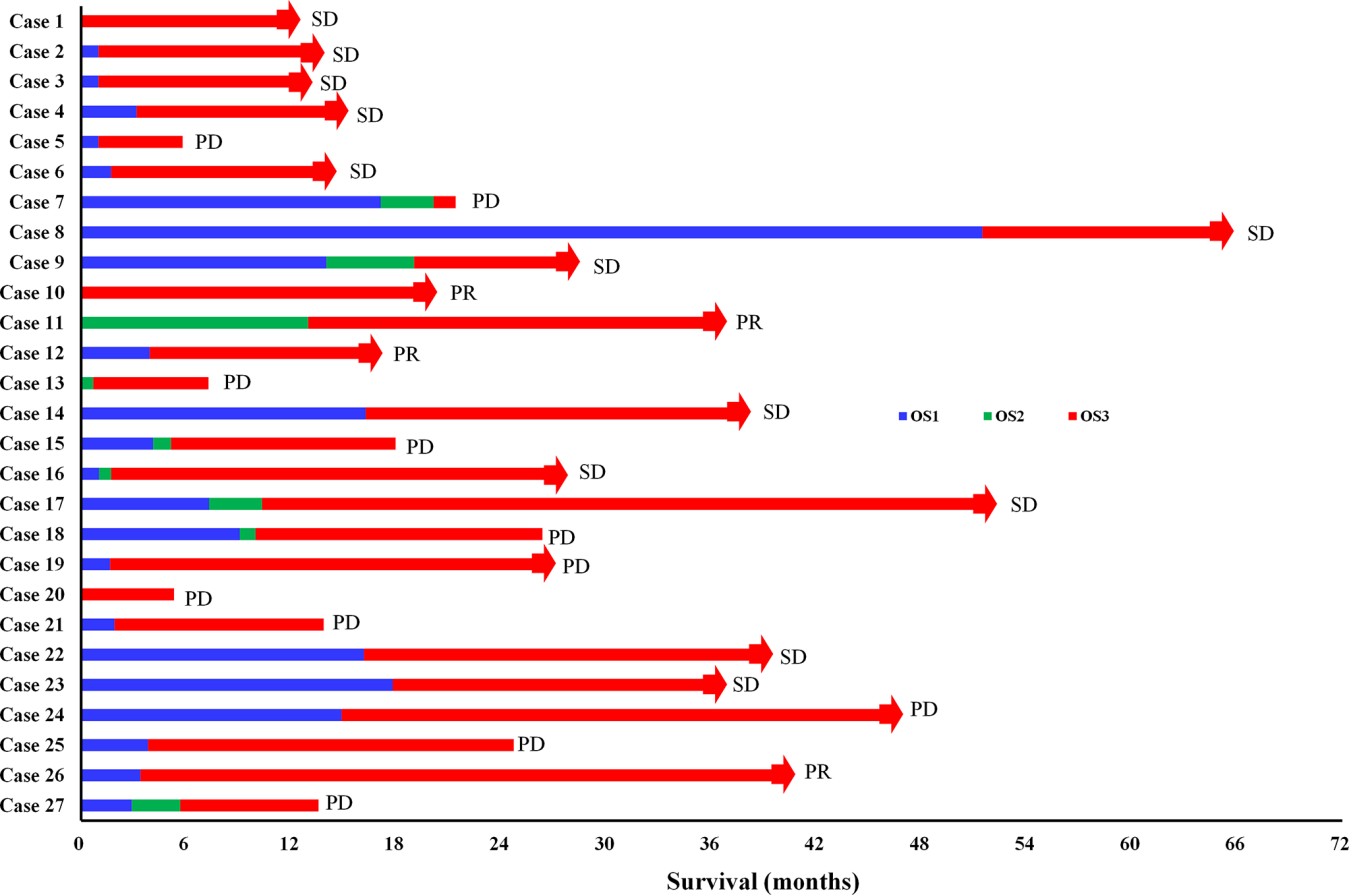
The treatment process of 27 DMPM patients. OS1: the time interval from surgery or chemotherapy to death or disease progression (PD); OS2: the time interval from second-line treatment to death or PD; OS3: the time interval from apatinib treatment to death or PD; CR, complete response; PR, partial response; SD, stable disease; The red bars with square end indicate patient death, and those with arrow end indicate patient still alive on apatinib treatment.

The treatment process of 27 DMPM patients were shown in **Figure 4**. Among these patients, the first-line treatment was CRS+HIPEC or pemetrexed plus platinum chemotherapy, second-line treatment was gemcitabine plus platinum, immunotherapy or other treatments, and third-line treatment was apatinib.

### A Typical Example of DMPM With Long SD After Apatinib Treatment

In April 2017, a 55-year-old non-smoking female patient was diagnosed as DMPM, with a disease history of abdominal distension for 10 months. Ultrasonography revealed multiple solid masses in the abdominal and pelvic cavity, greater omentum thickening, and large amount of ascites. Ultrasound-guided biopsy of the omentum revealed epithelioid malignant mesothelioma.

**Figure 5:**
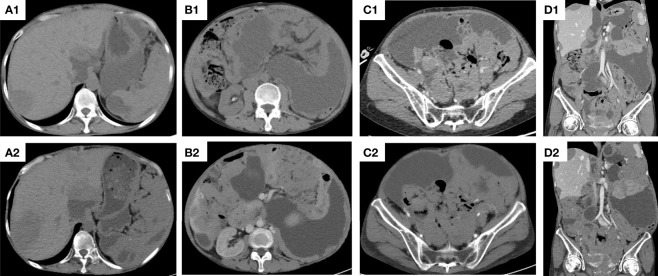
The computed tomography (CT) of the patient. **(A1)** Upper abdominal CT scan before apatinib treatment; **(A2)** Upper abdominal CT scan after apatinib treatment for 3 years; **(B1)** Lower abdominal CT scan before apatinib treatment; **(B2)** Lower abdominal (CT) scan after apatinib treatment for 3 years; **(C1)** Pelvic CT scan before apatinib treatment; **(C2)** Pelvic CT scan after apatinib treatment for 3 years; **(D1)** Coronal CT scan before apatinib treatment; **(D2)** Coronal CT scan after apatinib treatment for 3 years.

On May 12, 2017, the patient received CRS + HIPEC and again diagnosed as epithelioid type of DMPM. From July 12, 2017, the patient received pemetrexed plus cisplatin chemotherapy and bevacizumab targeted therapy. On February 6, 2018, the patient developed resistance to chemotherapy. Computed tomography (CT) on May 4, 2018 revealed cystic density shadows scattered in the abdominal and pelvic cavity and retroperitoneum, extensively compressing adjacent organs, and nodular soft tissue masses in the retro-peritoneum and the pelvic cavity (**Figures 5A1-D1**).

**Figure 6:**
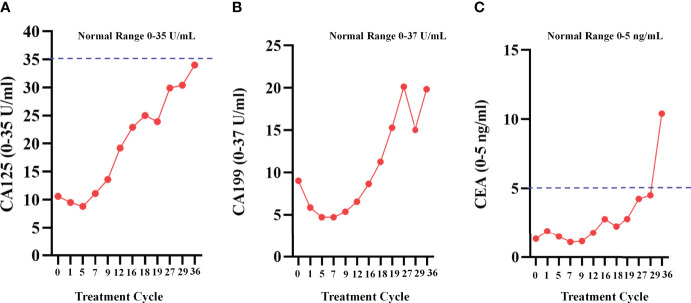
Serum tumor makers during apatinib treatment. **(A)**The change curve of CA125, during apatinib treatment, the values were normal; **(B)**The change curve of CA199; **(C)** The change curve of CEA.

**Figure 7:**
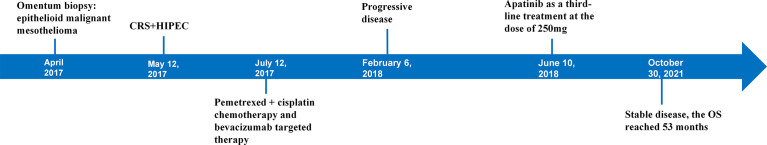
Patient treatment flowchart. CRS+HIPEC, Cytoreductive surgery plus hyperthermic intraperitoneal chemotherapy; OS, overall survival.

As the patient developed resistance to standard chemotherapy, on May 9, 2018, the patient was given apatinib as a third-line treatment at the dose of 250 mg/d. The patient tolerated the treatment well. At the most recent follow-up on June 18, 2021 (**Figures 5A2-D2**), the patient kept SD. During the apatinib treatment, the serum tumor makers of the patient was shown in **Figure 6**. The CA125 values and CA199 values were normal. At the time of this manuscript writing, the patient was still on apatinib maintenance therapy. By October 30, 2021, the OS of this patient after surgery has reached over 53 months (**Figure 7**).

### Adverse Events Analysis

Among the 27 DMPM patients, the common AEs were hypertension (6/27; 22.2%), hand-foot syndrome (5/27; 18.5%), albuminuria (4/27; 14.8%), anemia (4/27; 14.8%), leukopenia (4/27; 14.8%), rash (2/27; 7.4%), fatigue (2/27; 7.4%), oral ulcers (2/27; 7.4%), hoarseness (2/27; 7.4%), nausea/vomiting (2/27; 7.4%), diarrhea (2/27; 7.4%), headache (1/27; 3.7%), and fever (1/27; 3.7%). In terms of severity, the incidence rate of grade III/IV AEs was 16.2%. There was no grade V AEs (**Table 4**).

**Table 4:** Adverse events analysis of the 27 DMPM patients treated with apatinib.

Adverse events	Grade I	Grade II	Grade III	Grade IV	Total n (%)
Hypertension	4	1	1	0	6 (22.2)
Hand-foot syndrome	1	2	1	1	5 (18.5)
Albuminuria	2	2	0	0	4 (14.8)
Anemia	2	2	0	0	4 (14.8)
Leukopenia	1	3	0	0	4 (14.8)
Rash	0	1	1	0	2 (7.4)
Fatigue	0	1	1	0	2 (7.4)
Oral ulcers	1	1	0	0	2 (7.4)
Hoarseness	0	1	1	0	2 (7.4)
Nausea/vomiting	0	2	0	0	2 (7.4)
Diarrhea	0	2	0	0	2 (7.4)
Headache	0	1	0	0	1 (3.7)
Fever	0	1	0	0	1 (3.7)

DMPM, diffuse malignant peritoneal mesothelioma; n, number.

## Discussion

This study systematically evaluated the safety and efficacy of apatinib on refractory DMPM. The results indicated that apatinib could be an effective option for DMPM patients resistant to standard treatment.

Although the recommended chemotherapy regimen for DMPM is the combination of pemetrexed with cisplatin (17, 18), the efficacy is limited. Moreover, the efficacy and safety of targeted therapy and immunotherapy for DMPM are less clear. VEGF inhibitor bevacizumab has been explored as a therapeutic option for malignant pleural mesothelioma, however the relevant clinical trials of VEGF inhibitors in DMPM are few (19). A recent study indicated that the combination of programmed death ligand 1 (PD-L1; atezolizumab) with VEGF blockade (bevacizumab) was well-tolerated and led to robust and durable responses in DMPM patients who had progressed on or were intolerant to prior platinum-pemetrexed chemotherapy.

Apatinib is a multi-target small molecule tyrosine kinase inhibitor. The functions of apatinib include not only inhibiting neovascularization, but also directly restraining tumor cells proliferation, migration or inducing apoptosis. Evidence showed that apatinib inhibited the migration of ovarian cancer cells SKOV3 and HO8910, and effectively inhibited tumor growth *in vivo* (11). Deng et al. reported that apatinib distinctly inhibited cell growth and promoted apoptosis in both B and T lineage ALL cell lines in a dose- and time-dependent manner (20). Our previous studies showed that apatinib immediately inhibited DMPM cells proliferation by intervening cells cycle (15, 16), In addition, Du et al. reported a case of apatinib treated for epithelioid malignant pleural mesothelioma (21). The patient was resistant to pemetrexed/gemcitabine combined with cisplatin. Apatinib was used as a third-line regimen and disease-free survival of the patient was up to 5 months. The AEs included hand-foot syndrome, proteinuria and hypertension. These data suggest that apatinib is safe and effective for DMPM. Furthermore, apatinib could be more suitable for DMPM patients in view of the characteristics with affordable cost, oral administration, and no need to be hospitalized.

Relevant studies on apatinib for the treatment of DMPM are few. Our pre-clinical and clinical studies provided evidence for the treatment of DMPM with apatinib, and offered a new treatment option and idea for this orphan disease. However, there are some shortcomings in this study, as the following: (1) the small sample size pilot study limits the strength of the evidence level; (2) this study only suggests that apatinib is effective for epithelioid DMPM, it is necessary to explore the efficacy for biphasic and sarcomatous DMPM; (3) more treatment patterns and molecular mechanisms should be further investigated.

In conclusion, this pilot study indicated that apatinib could be effective for epithelioid DMPM. We will conduct further clinical studies to clarify the efficacy and safety of apatinib, and explore multiple treatment modalities, such as neoadjuvant or adjuvant chemotherapy combined with apatinib, and apatinib maintenance therapy. More importantly, our preclinical research has showed that apatinib inhibited DMPM cell proliferation by intervening cells cycles, and the relevant molecular mechanisms should be further investigated.

## Data Availability Statement

The original contributions presented in the study are included in the article/supplementary material. Further inquiries can be directed to the corresponding author.

## Ethics Statement

All experiments were performed under the guideline of the Scientific Research Ethics Committee of Beijing Shijitan Hospital, Capital Medical University [Approval number: 2020 Research Ethics Review No. (2)]. The patients/participants provided their written informed consent to participate in this study. Written informed consent was obtained from the individual(s) for the publication of any potentially identifiable images or data included in this article.

## Author Contributions

ZR-Y was involved in study design, data collation, data analysis, research implementation and data interpretation, and manuscript writing. YD-S was involved in data collation and data analysis. HL-W and RM were contributed to data analysis, research implementation and data interpretation. YL was contributed to study design, data analysis and interpretation, design of figures. All authors were involved in manuscript development, did a full review of the article, have approved the final version of the manuscript, and are responsible for all content.

## Funding

The work was funded by Beijing Municipal Administration of Hospitals’ Ascent Plan (DFL20180701); Beijing Municipal Grant for Medical Talents Group on Peritoneal Surface Oncology (2017400003235J007); National Natural Science Foundation of China (82073376).

## Conflict of Interest

The authors declare that the research was conducted in the absence of any commercial or financial relationships that could be construed as a potential conflict of interest.

## Publisher’s Note

All claims expressed in this article are solely those of the authors and do not necessarily represent those of their affiliated organizations, or those of the publisher, the editors and the reviewers. Any product that may be evaluated in this article, or claim that may be made by its manufacturer, is not guaranteed or endorsed by the publisher.
